# Neutralizing Monoclonal Antibodies against the Gn and the Gc of the Andes Virus Glycoprotein Spike Complex Protect from Virus Challenge in a Preclinical Hamster Model

**DOI:** 10.1128/mBio.00028-20

**Published:** 2020-03-24

**Authors:** James Duehr, Meagan McMahon, Brandi Williamson, Fatima Amanat, Alan Durbin, David W. Hawman, Danny Noack, Skyler Uhl, Gene S. Tan, Heinz Feldmann, Florian Krammer

**Affiliations:** a Department of Microbiology, Icahn School of Medicine at Mount Sinai, New York, New York, USA; b Graduate School of Biomedical Sciences, Icahn School of Medicine at Mount Sinai, New York, New York, USA; c Laboratory of Virology, Division of Intramural Research, Rocky Mountain Laboratories, National Institute of Allergy and Infectious Diseases, National Institutes of Health, Hamilton, Montana, USA; d Infectious Diseases, The J. Craig Venter Institute, La Jolla, California, USA; e Department of Medicine, University of California San Diego, La Jolla, California, USA; St. Jude Children's Research Hospital

**Keywords:** Andes virus, hantavirus, MAb, Sin Nombre virus

## Abstract

Hantaviruses are the etiological agent of hemorrhagic fever with renal syndrome (HFRS) and hantavirus cardiopulmonary syndrome (HCPS). The latter is associated with case fatality rates ranging from 30% to 50%. HCPS cases are rare, with approximately 300 recorded annually in the Americas. Recently, an HCPS outbreak of unprecedented size has been occurring in and around Epuyén, in the southwestern Argentinian state of Chubut. Since November of 2018, at least 29 cases have been laboratory confirmed, and human-to-human transmission is suspected. Despite posing a significant threat to public health, no treatment or vaccine is available for hantaviral disease. Here, we describe an effort to identify, characterize, and develop neutralizing and protective antibodies against the glycoprotein complex (Gn and Gc) of Andes virus (ANDV), the causative agent of the Epuyén outbreak. Using murine hybridoma technology, we generated 19 distinct monoclonal antibodies (MAbs) against ANDV GnGc. When tested for neutralization against a recombinant vesicular stomatitis virus expressing the Andes glycoprotein (GP) (VSV-ANDV), 12 MAbs showed potent neutralization and 8 showed activity in an antibody-dependent cellular cytotoxicity reporter assay. Escape mutant analysis revealed that neutralizing MAbs targeted both the Gn and the Gc. Four MAbs that bound different epitopes were selected for preclinical studies and were found to be 100% protective against lethality in a Syrian hamster model of ANDV infection. These data suggest the existence of a wide array of neutralizing antibody epitopes on hantavirus GnGc with unique properties and mechanisms of action.

## Abstract (precis)

Infections with New World hantaviruses are associated with high case fatality rates, and no specific vaccine or treatment options exist. Furthermore, the biology of the hantaviral GnGc complex, its antigenicity, and its fusion machinery are poorly understood. Protective monoclonal antibodies against GnGc have the potential to be developed into therapeutics against hantaviral disease and are also great tools to elucidate the biology of the glycoprotein complex.

## INTRODUCTION

**FIG 1:**
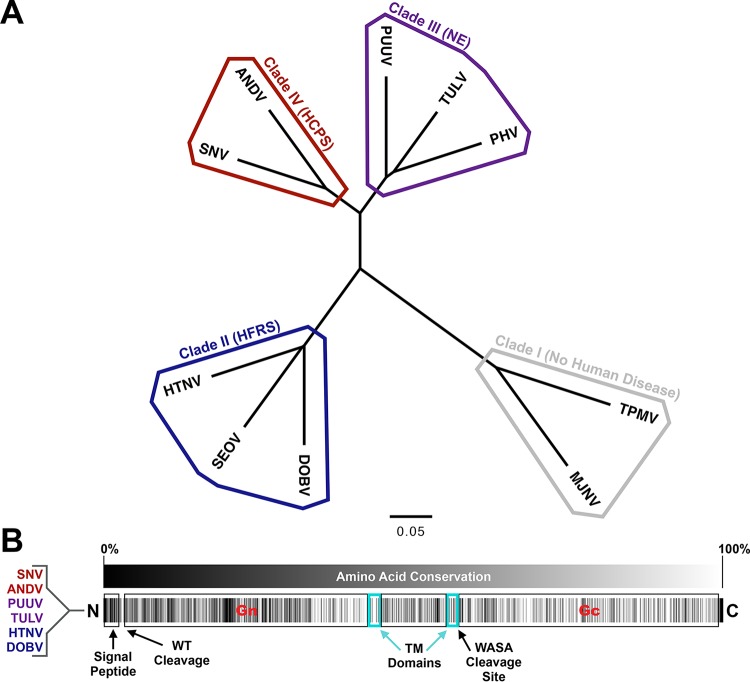
Phylogenetic tree and conservation analysis of the *Hantaviridae* family. (A) Phylogenetic tree of M segments from hantavirus clades I to IV based on amino acid sequences shown in Table S1 in the supplemental material. ANDV, Andes virus; DOBV, Dobrava-Belgrade virus; HTNV, Hantaan virus; MJNV, Imjin virus; PHV, Prospect Hill virus; PUUV, Puumala virus; SEOV, Seoul virus; SNV, Sin Nombre virus; TPMV, Thottapalayam virus; TULV, Tula virus. TPMV and MJNV (thottimviruses) are included as part of an outgroup. The scale bar represents 5 variants per 100 amino acids. (B) Conservation map of the M segment of the *Orthohantavirus* genus based on amino acid sequences from Table S1. Transmembrane (TM) domains are based upon analysis via TMHMM. Protease cleavage sites for the signal peptidase are indicated (including the conserved WAASA motif). Black color indicates less conserved and white color indicates more conserved residues.

Collectively, *Hantaviridae* family members are responsible for approximately 200,000 human illnesses each year (1). Of these, the vast majority are cases of hemorrhagic fever with renal syndrome (HFRS) or nephropathia epidemica (NE), a milder form of HFRS. A small minority (around 300) are cases of hantavirus cardiopulmonary syndrome (HCPS). The viruses that cause these three diseases are often classified into clades II, III, and IV, based on M segment sequence diversity (Fig. 1A). Viruses of clade I, including *Thottapalayam virus* and *Imjin virus*, are not known to cause any disease in humans (2).

10.1128/mBio.00028-20.6 TABLE S1 Amino acid sequences used for the construction of Fig. 1. Download Table S1, DOCX file, 0.1 MB. Copyright © 2020 Duehr et al. 2020 Duehr et al. This content is distributed under the terms of the Creative Commons Attribution 4.0 International license.

Despite their relatively low incidence rate, HCPS viruses remain pathogens of importance in military medicine, due to their widespread availability in wild hosts throughout rural areas worldwide (3). Hantaviruses are theorized to be transmitted via aerosolization of feces or urine (2, 4). In addition, human-to-human transmission of Andes virus (ANDV) has long been suspected, based upon case reports and contact tracing in the days following intermittent outbreaks. Notably, a recent outbreak in the southwestern province of Chubut in Argentina has underscored the increasing size and frequency of HCPS flare ups in recent years. Between October 2018 and February of 2019, the total number of hantavirus cases detected was 4-fold higher than the number typically seen in the whole of Argentina in twelve months (5). When a spike in cases was first recorded, the public health agency acted quickly to institute a 110-person quarantine in Epuyén, the epicenter of the outbreak (6). Importantly, such quarantine measures are not typically used during HFRS or HCPS outbreaks. With confirmed infection in at least 31 individuals and at least 11 deaths, this could also be one of the biggest recorded ANDV outbreaks (7). Importantly, human-to-human transmission is strongly suspected to have played a significant role in the Epuyén outbreak (7).

The most diverse region of the hantavirus genome (8), which tracks directly to host/organ tropism (9), is the M segment that codes for GnGc. More specifically, M segment mRNA is translated by host machinery into a precursor protein that is cotranslationally cleaved into Gn and Gc components, which then associate on the virion membrane during packaging (10–12). GnGc can bind to and achieve entry via host β-3-integrin, decay accelerating factor, complement receptor gC1qR-p32, and protocadherin-1 (11, 13–16). However, other than the putative Gc fusion loop (17) and the known contacts between Gn and Gc (12), it is not known which portions of Gn or Gc are involved in any of these interactions (18). Aside from the acidification model of Hantaan virus (HTNV) GnGc (10, 11), exactly how the ANDV Gn and Gc cooperate to achieve attachment/entry remains unclear (19).

Further characterization of these proteins and their relationship to disease is heavily predicated on understanding their antigenic nature. Likewise, vaccine and therapeutic design rely heavily on an understanding of how a pathogen presents itself to the immune system (20). One method of determining these interactions uses monoclonal antibodies (MAbs) as both epitope mapping agents and tools in structural/functional characterization (21–23). Unfortunately, MAbs, especially neutralizing and protective MAbs, are severely lacking in the hantavirus field. In a recent study conducted by Garrido et al., two anti-ANDV GnGc MAbs were successfully tested for therapeutic efficacy in animal models in a cocktail setting (24), clearly demonstrating that anti-GnGc MAbs may be useful in combatting this disease. However, the range of possible properties such MAbs may take has yet to be fully explored. No anti-ANDV MAbs have thus far been evaluated for mechanism of action or for protection on an individual basis. It is unclear what diversity of functions such antibodies possess or even where they bind on the ANDV GnGc. If the history of therapeutic MAb development in the emerging ebolaviruses is any indication, multiple distinct epitope-binding and functionally distinct MAbs will be necessary to develop effective MAb therapies for human use (25–27).

Here, we describe an effort to generate and characterize a panel of MAbs raised against the ANDV GnGc complex. These MAbs are diverse with respect to a number of characteristics, including mechanism of action, neutralization, binding site, epitope character, and *in vivo* protection. In further characterizing the structural mode of interaction these MAbs have with the ANDV GnGc, we hope to provide a road map for future vaccination strategies but also a set of neutralizing and protective epitopes on the ANDV glycoproteins that will drive future investigations into fundamental biological mechanisms of host-virus interaction. Given the 100% protection the four tested MAbs provided in the Syrian hamster model, these MAbs may also serve well as components of future therapies for the prevention and treatment of HCPS in humans.

(The data in this paper were used by James Duehr in a dissertation in partial fulfillment of the requirements for a PhD degree at the Graduate School of Biomedical Sciences at Mount Sinai, New York, NY, 2019.)

## RESULTS

### Vaccination strategy and hybridoma fusion.

To generate a set of MAbs with diverse activity against ANDV GnGc, we used two distinct murine vaccination regimens (to induce antibodies against Gn and Gc, respectively). Both strategies involved a prime-boost regimen, specifically, DNA vaccines expressing GnGc of one of several hantavirus species, followed by a boost with a live vesicular stomatitis virus (VSV) expressing the Andes virus glycoprotein spike complex in place of the wild-type VSV glycoprotein (VSV-ANDV) (28). Each vaccination given to the BALB/c mice was separated by an interval of 4 weeks in order to allow for proper affinity maturation and generation of memory B-cells (29, 30).

The first immunization strategy involved priming with ANDV DNA plasmids expressing full-length GnGc, followed by immunizations with VSV-ANDV to drive a highly specific ANDV Gn-focused antibody response (AN fusion). This is based on preliminary structural studies suggesting that Gc is shielded by and therefore likely immunosubdominant to the more prominently accessible Gn (18, 19). We further hypothesized that a heterologous priming strategy could drive antibody generation against Gc. The hantavirus Gn is also considerably more genetically diverse than Gc (Fig. 1B), and so heterologous priming would perhaps avoid secondary B cell responses against Gn and instead drive antibodies toward the more conserved Gc, consistent with long-held models of original antigenic sin/imprinting that have been proposed for influenza virus (31–34). To this end, a second strategy was devised involving the cross-priming of mice with plasmids coding for HTNV GnGc (in clade II of hantavirus phylogeny), then ANDV GnGc (clade IV), and finally, Puumala virus (PUUV) GnGc (clade III). Mice were then boosted with VSV-ANDV as described above (HAP fusion). An overview of both vaccination strategies and the subsequent screening process is presented in Fig. S1 in the supplemental material. After the last boost, splenocytes were harvested and fused with the Sp2/0-Ag14 mouse myeloma cell line to form murine hybridomas (35). The resulting hybridoma clones were then then screened via enzyme-linked immunosorbent assay (ELISA) and immunostaining against Vero.E6 cells infected with either VSV-ANDV or wild-type VSV (Indiana strain) to select for antibodies that bind to ANDV GnGc but not VSV proteins or host proteins present in infected cells. Overall, of 17 specific IgG MAbs that were isolated, eight arose from the homologous prime-boost vaccination regimen (AN) and nine from the heterologous regimen (HAP) (Fig. S1).

10.1128/mBio.00028-20.1 FIG S1 Vaccination regimens and hybridoma screening statistics. (A) Two groups of five female BALB/c mice each were vaccinated with the given DNA harbored by pCAGGS mammalian expression plasmids in two or three separate events, 100 μg each, separated by 21-day intervals. DNA vaccinations were done via intramuscular (i.m.) injection followed by electroporation at the injection site. Subsequent boost(s) were composed of 10^5^ PFU VSV-ANDV injected i.p. Only the mouse used for hybridoma fusion was given the final boost. Three days after the last boost, the mouse was anesthetized and the spleen excised for use in a hybridoma fusion. (B) Diagram showing the statistics of the ensuing two hybridoma fusions. Of 1,729 picked clones, 11.91% were positive for VSV-ANDV and negative for VSV-WT via ELISA and immunostaining of infected cells. Of 206 clones in the aforementioned category (VSV-ANDV[+])/VSV-WT[−]), 19 were monoclonal IgG per rapid ELISA isotyping. Of those 19, 2 were IgG1, 12 were IgG2a, and 5 were IgG2b. These 19 clones were used for subsequent assays. Download FIG S1, DOCX file, 0.2 MB. Copyright © 2020 Duehr et al. 2020 Duehr et al. This content is distributed under the terms of the Creative Commons Attribution 4.0 International license.

### Characterization of antibody binding.

**FIG 2:**
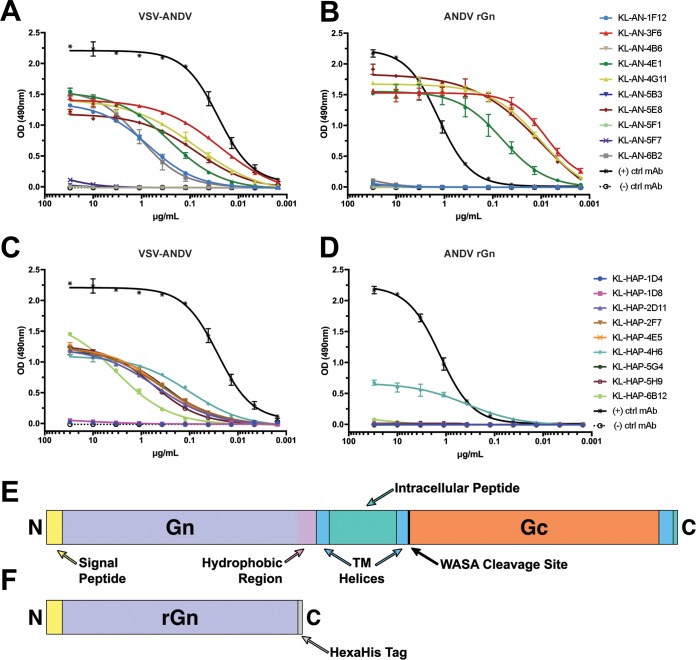
ELISA reactivity against purified VSV-ANDV and rGn. (A) ELISAs of MAbs from the homologous “AN” hybridoma fusions against VSV-ANDV. Plates were coated with 5 μg/ml of purified VSV-ANDV, while MAbs were used in 1:3 serial dilutions beginning with 30 μg/ml. Data shown are the products from two replicates. The positive control was an anti-VSV-N MAb and common between panels A and C. The negative control was KL-2G12, an IgG2a MAb against Zaire ebolavirus GP. All trend lines are logarithmic regressions except where such regressions were not converged; in such cases, connecting lines were used. (B) ELISAs against recombinant ANDV Gn. The recombinant ANDV Gn was produced in insect cells via baculovirus expression. Plates were coated with 2 μg/ml of ANDV rGn, while MAbs were used in 1:3 serial dilutions beginning with 30 μg/ml. Data shown are the products from two separate experiments, two replicates each. The positive control was an anti-hexahistidine tag antibody and common between panels B and D. Negative control was KL-2G12, an IgG2a MAb against Zaire ebolavirus GP. (C) ELISAs conducted against VSV-ANDV as for panel A but with MAbs sourced from the heterologous “HAP” hybridoma fusions as described for Fig. S1. (D) ELISAs conducted against ANDV rGn as for panel B but with MAbs sourced from HAP fusions as described for Fig. S1. (E) Schematic of full GnGc with features indicated. (F) Schematic of recombinant Gn used in panels B and D. Transmembrane domains, the signal peptide, and the Gn hydrophobic region were annotated based on TMHMM analysis. Both schematics are to scale.

The specificity of clones for the intended antigen (and not the vector itself) is an important factor, but it provides little to no information about the character of binding. To more accurately characterize the binding of these MAbs, we also performed ELISAs in dilution series against the mature GnGc as presented on the surface of purified VSV-ANDV particles (Fig. 2A and C). From these ELISAs, it can be seen that the majority of MAbs specifically reactive to VSV-ANDV-infected Vero cells were also reactive to purified VSV-ANDV virions (12/19). MAbs which reacted to infected cells but not purified virus may perhaps target epitopes available in GnGc postfusion or during maturation through the endoplasmic reticulum (ER) and Golgi. No MAbs reacted to purified wild-type VSV (see Fig. S2).

10.1128/mBio.00028-20.2 FIG S2 ELISAs against purified wild-type VSV. ELISAs of MAbs from AN (A) and HAP (B) fusions against VSV-WT. Experiments were conducted as described for Fig. 2A and C, with shared positive and negative controls. Download FIG S2, DOCX file, 0.2 MB. Copyright © 2020 Duehr et al. 2020 Duehr et al. This content is distributed under the terms of the Creative Commons Attribution 4.0 International license.

To broadly determine epitope localization, we performed ELISAs against a recombinant soluble version of the ANDV Gn (Fig. 2B and D), expressed using the baculovirus system in insect cells (36). A schematic of recombinant Gn (rGn) compared to the full-length GnGc is provided in Fig. 2E and F. All areas noted as transmembrane domains were predicted using TMHMM (37). Overall, five MAbs bound to recombinant Gn, 4/10 (KL-AN-3F6, KL-AN-4E1, KL-AN-4G11, and KL-AN-5E8) from the homologous immunization scheme (AN) and 1/9 (KL-HAP-4H6) from the heterologous scheme (HAP). The recombinant Gn used here was shortened to remove the hydrophobic region at the C terminus, which is likely involved in association between Gn, Gc, and the membrane. This hydrophobic region could, therefore, make protein expression more difficult. MAbs which do not bind this recombinant Gn could bind to the Gc or to the glycoprotein complex only in the presence of both Gn and Gc.

ELISA provides useful binding data, but it does not provide much information about the character of epitopes. To assess whether the epitope of each MAb is conformational or linear, we used Western blots (WBs) against VSV-ANDV-infected cells (see Fig. S3). This also allowed us to assess further whether each MAb may bind Gn or Gc, based on the apparent size of protein bands. If a MAb bound in an ELISA (Fig. 2) but not in a Western blot under any condition, we interpreted the epitope as “conformational.” If a MAb bound in a Western blot under any condition, we interpreted the epitope as “linear” or, at the very least, “microconformational.” Interestingly, four MAbs bound the virus under nonreducing conditions, suggesting a linear or microconformational (e.g., features that refold on the blot during incubation in buffer) epitope (Fig. S3A), but one MAb (KL-AN-4H6) lost reactivity under reducing conditions (incubated with β-mercaptoethanol [BME]) (Fig. S3B). It is possible this conformational character of 4H6 binding is due to contacts on the ANDV glycoprotein that are separated upon BME-mediated disulfide bond reduction. In reactive MAb blots, there were two distinct band sizes in positive blots (∼28 kDa and ∼50 kDa). Based on the ELISA data for reactivity of each MAb to our rGn construct, these bands might represent linearized ANDV Gn and Gc, respectively. There is a size discrepancy in Gn, which may be the result of cleavage from cellular proteases.

10.1128/mBio.00028-20.3 FIG S3 Western blots against lysates of cells infected with VSV-ANDV. Vero.E6 cells were infected with an MOI of 1 of VSV-ANDV or influenza virus (A/Netherlands/602/2009 [H1N1]) and harvested after 48 h of incubation at 37°C. Cells were then resuspended in NP-40 lysis buffer and combined 1:1 with Laemmli buffer either with (A) or without (B) BME (β-mercaptoethanol). Lysates were then run on a 2% to 10% gradient SDS-PAGE gels and blotted with each MAb at 30 μg/ml as a primary stain. Secondary stain was anti-mouse alkaline phosphatase-conjugated antibody (1:1,000) and development was via AP development system (Bio-Rad). The ladder is based upon a color protein standard (Life Technologies). Download FIG S3, DOCX file, 0.6 MB. Copyright © 2020 Duehr et al. 2020 Duehr et al. This content is distributed under the terms of the Creative Commons Attribution 4.0 International license.

### Neutralization and Fc effector functions.

**FIG 3:**
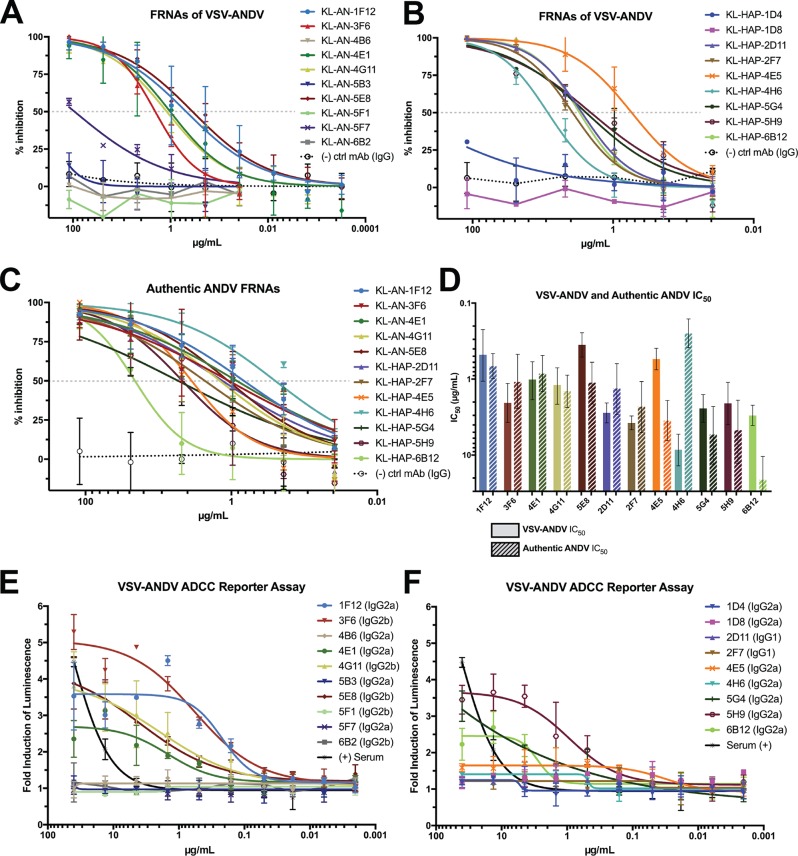
Neutralization and effector functions of the isolated MAbs. (A) FRNAs of “AN” fusion MAbs against VSV-ANDV. (B) FRNAs of “HAP” fusion MAbs against VSV-ANDV. For both A and B, each MAb was run in duplicates in 3-fold serial dilutions starting at 30 μg/ml. Any MAb exhibiting >15% neutralization at the last concentration was repeated with lower dilutions. The negative-control MAb was a murine IgG2a specific for Zaire ebolavirus (KL-2G12). All trend lines are logarithmic regressions except where such regressions were not converged. (C) FRNAs conducted as for panels A and B but against authentic ANDV. Dashed lines in panels A, B, and C demonstrate 50% inhibition. (D) Comparison of IC_50_ values of each MAb against VSV-ANDV and authentic ANDV. Error bars indicate 95% confidence intervals. (E and F) ADCC reporter assays of each MAb against VSV-ANDV-infected Vero.E6 cells (MOI, 1.0). Data shown are the result from one experiment with a shared positive control in panels E and F (serum from homologous fusion). This positive control was used in 3-fold serial dilutions with a starting dilution of 1:300.

Binding capacity is important, especially for diagnostic assays, but it does not always correlate with functionality or protective efficacy. In a large-scale assessment of protection in anti-ebolavirus MAbs, the most effective predictor (although not absolute) of protection was neutralization (27). To explore whether our MAbs could neutralize viral particles via binding to GnGc *in vitro*, we used focus-forming unit reduction neutralization assays (FRNAs) against VSV-ANDV (Fig. 3A and B). Interestingly, each MAb that bound purified VSV-ANDV in an ELISA was also neutralizing via FRNA, regardless of rGn reactivity in an ELISA/WB. Likewise, we wanted to verify that each MAb neutralized the authentic ANDV, as it is possible, though unlikely, that neutralizing epitopes on the authentic virus are expressed in an altered fashion on VSV-ANDV. However, all neutralizing MAbs against VSV-ANDV were also able to neutralize authentic ANDV (Fig. 3C). A comparison of 50% inhibitory (IC_50_) values against VSV-ANDV and authentic ANDV (Fig. 3D) demonstrated that the neutralizing activity was similar for most MAbs.

While neutralization is a very effective predictor (and mechanism) of protection for many viruses, it is not the only such predictive factor. Another useful mechanism by which antibodies can protect against infection are antibody-mediated effector functions. The Fc regions of certain antibodies have been shown to engage the FcRs of lymphocytes and precipitate the killing or phagocytosis of infected cells and/or virions (38). In particular, the process of antibody-dependent cellular cytotoxicity (ADCC) is mediated by natural killer (NK) cells, which are upregulated in survivors of hantavirus infection (39).

There are well-characterized reporter assays to assess the potential of a MAb to stimulate ADCC activity (40, 41). When assessed in a bioluminescent reporter assay (Fig. 3E and F), these MAbs recapitulated some known murine IgG subtype trends. Typically, IgG2a stimulates the most ADCC activity, followed by IgG2b, while IgG1 exhibits the least activity, and this observation is consistent with the data shown here. Another trend that has been explored is the influence of epitope location on ADCC activity. For influenza A viruses, antibodies directed against the stalk of hemagglutinin tend to stimulate more effector function activity and require very specific epitope contacts (42). For Zaire ebolavirus, the opposite is observed: MAbs binding near the tip of the glycoprotein (GP) having more FcR activity than antibodies binding closer to the membrane (27). Our data represents a middle ground in the case of antibodies directed against the hantavirus GnGc: all of our non-IgG1 anti-ANDV neutralizing MAbs (KL-AN-1F12, KL-AN-3F6, KL-AN-4E1, KL-AN-4G11, and KL-AN-5E8) were ADCC active, regardless of epitope localization. The localization of our epitopes remains to be definitively confirmed via crystallization, but this represents an interesting aspect of GnGc biology if confirmed.

### Epitope characterization via escape mutagenesis of VSV-ANDV.

**FIG 4:**
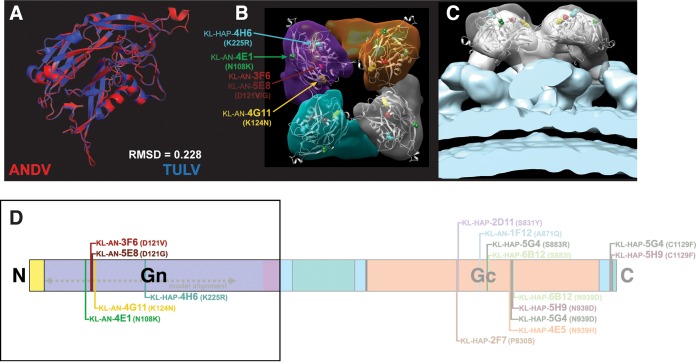
Visualizing VSV-ANDV escape mutations on a computationally fit model of ANDV Gn. A model of ANDV Gn was created using TULV Gn as the template (PDB 5FXU). This model was then computationally fit into a TULV cryo-EM tomograph to depict the relationship between the Gn and the plasma membrane. (A) Structural alignment of ANDV model (red) and TULV Gn structure (blue). (B) Top view of the glycoprotein complex with escape mutations visualized on the model using colors as indicated. The tetramer is the assumed arrangement for ANDV, confirmed for TULV. (C) Side view including the plasma membrane shown in blue. (D) To-scale schematic of the ANDV GnGc showing the location of escape mutations and the alignment sequence used for structural modeling. RMSD, root mean square deviation.

The ability of MAbs to neutralize virus also allows for the generation of escape mutations which can provide information about epitope location. Via copassaging of VSV-ANDV with an antibody, followed by sequencing of each clonally isolated mutant, we identified amino acid positions important for the binding of each neutralizing MAb. We then visualized these mutations on a computational model of the ANDV Gn (Fig. 4A to C). This model was based upon a cryo-electron microscopy (EM)-derived structure published based on Tula virus (TULV), a different hantavirus in clade III (PDB 5FXU). The model was deemed useful for analysis based upon the relatively high level of similarity between TULV and ANDV Gn, in terms of sequence and structure (73.8% sequence similarity per EMBOSS-Needle; 0.228 root mean square deviation [RMSD] of structural fit) (Fig. 4C). Using Chimera, a computational fit of this ANDV Gn model into a cryo-electron tomograph of the TULV envelope published by Li et al. was then performed (43). To that end, a predetermined mapping correlation cutoff was set at 0.8. Two distinct fits were above the cutoff; the more well-correlated of the two was also the most similar to the fit of TULV Gn performed by Li et al., and so this is the positioning shown in Fig. 4. With this most-favorable computational fit, all mutated amino acid positions in escape mutants of Gn-binding MAbs are positioned on the external surface of the Gn, distal to the membrane in each monomer of the heterotetrameric spike complex (KL-AN-3F6, N121D; KL-AN-4E1, N108K; KL-AN-4G11, K124N; KL-AN-5E8, N121G; KL-HAP-4H6, K225R). A full listing of the names and locations of escape mutants on the full GnGc is provided on the GnGc schematic in Fig. 4D. Interestingly, a survey of these results showed that there are some MAbs which induced overlapping and multivariant escape mutations on the Gc (KL-HAP-4E5, KL-HAP-5G4, KL-HAP-5H9, and KL-HAP-6B12; KL-HAP-2D11 and KL-HAP-2F7). This may be indicative of immunodominant antigenic sites and has been observed in MAbs raised against HTNV and PUUV GnGc (44–48.)

**FIG 5:**
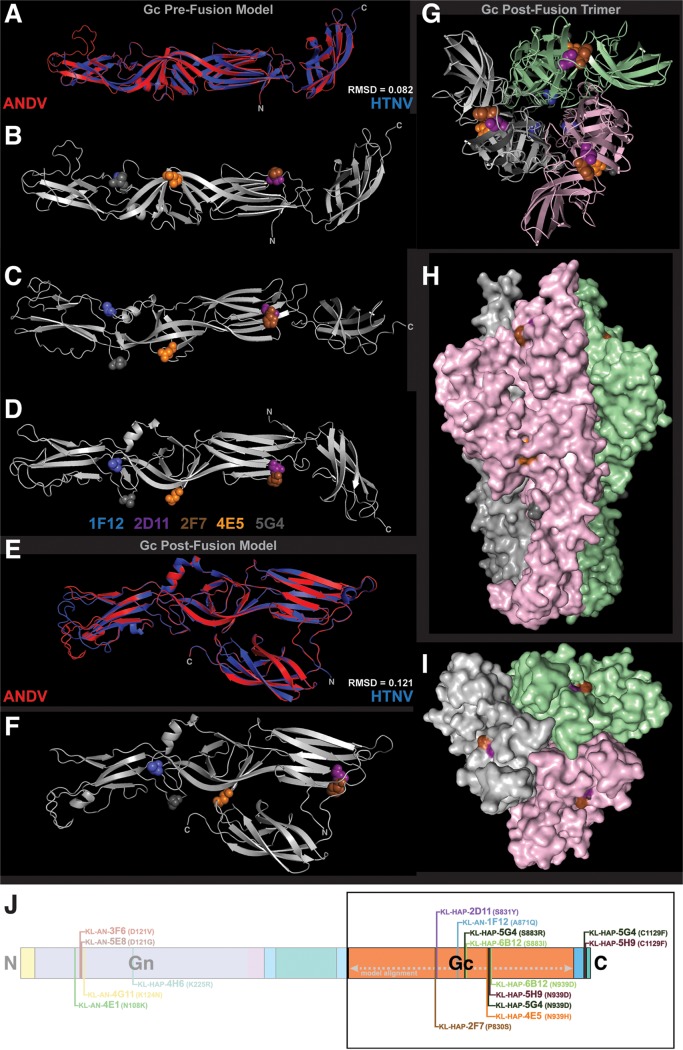
Visualizing VSV-ANDV escape mutations on a computational model of ANDV Gc. Models of ANDV Gc pre- and postfusion were created using HTNV Gc as the template (prefusion, PDB 5LJY; postfusion, PDB 5LK3). (A) Structural fit of the ANDV prefusion Gc model with 5LJY. (B to D) visualization of escape mutations on the prefusion ANDV Gc model. (E) Structural fit of the ANDV postfusion Gc model (monomer) with 5LK3. (F) Visualization of escape mutations on the postfusion Gc model as a monomer. (G to I) Visualization of escape mutations on the postfusion Gc model as a trimer. Colors shown below panel D are representative for all structures included here. (J) Schematic of ANDV GnGc with escape variants. RMSD, root mean square deviation.

To visualize escape mutations located on the VSV-ANDV Gc, computational models were constructed of the ANDV Gc in both pre- and postfusion conformations (Fig. 5), based upon crystal structures of these two conformations published by Guardado-Calvo et al. (PDB 5LJY and 5LK3, respectively) (49). The fidelity of these models is shown by the sequence and structural similarity of the models to their templates (77.1% similar and 64.9% identical per EMBOSS-Needle; RMSD = 0.082 and 0.121 for pre- and postfusion Gc, respectively). Figure 5A depicts this structural fit of the prefusion ANDV and HTNV Gc, while Fig. 5B to D show the escape mutations on the ANDV Gc prefusion monomer. The structural hinge regions of this molecule may play a role in how a similar TULV prefusion Gc fits into the narrow map segments that exist between the Gn and the membrane in the cryo-electron tomograph shown in Fig. 4B and C (49). Figure 5B to D and F depict escape mutations induced by MAbs on the VSV-ANDV Gc (KL-AN-1F12, A871Q; KL-HAP-2D11, S831Y; KL-HAP-2F7, P830S; KL-HAP-4E5, N939H; KL-HAP-5G4, S883R, N939D, and C1129F; KL-HAP-5H9, N939D and C1129F; KL-HAP-6B12, S883I and N939D). Figure 5E shows the structural fit between postfusion ANDV and HTNV Gc, and Fig. 5F shows the Gc monomer with escape mutations emphasized by color and side chain. Figure 5G to I depict the postfusion ANDV Gc in a trimeric complex based on the primary biological assembly of HTNV Gc published by Guardado-Calvo et al. (49) As is shown here and in the postfusion Gc monomer, it is unlikely that KL-AN-1F12 or KL-HAP-4E5 bind the postfusion ANDV Gc, as the crucial residues for binding are completely (KL-AN-1F12) or partially (KL-HAP-4E5) obscured in the trimer (Fig. 5I).

What this visual mapping demonstrates is the wide variety of epitopes targeted by these MAbs on the GnGc complex. These mutations also confirm that 5/6 neutralizing antibodies from the heterologous vaccination scheme (HAP) have contacts on the Gc surface. The reverse is true for the homologous scheme (AN): 5/6 of these MAbs have contacts on Gn. To confirm epitope mapping via deep sequencing, FRNAs of each MAb against each clonally isolated escape mutated virus were performed. The IC_50_ value of each MAb against each virus was visualized as a heat map (see Fig. S4). The complete set of FRNA data is available in Fig. S5. Based on both sequencing and cross-neutralization data, these 12 neutralizing MAbs bind 7 distinct epitopes spread across the surfaces of ANDV Gn and Gc. Several neutralizing MAbs have overlapping or interacting epitopes, including KL-AN-4G11 and KL-AN-5E8, KL-HAP-2D11 and KL-HAP-2F7, KL-HAP-4E5 and KL-HAP-6B12, and all four of KL-HAP-4E5, KL-HAP-5G4, KL-HAP-5H9, and KL-HAP-6B12.

10.1128/mBio.00028-20.4 FIG S4 Heat map of VSV-ANDV escape mutant IC_50_ values. Escape mutants were plaque purified to ensure monoclonal virus populations, before growth and titer determination. FRNAs of each neutralizing antibody against each escape virus were then conducted, to understand the relationship between epitopes and to confirm the escape phenotype. Full graphs of each FRNA are available in Fig. S5. IC_50_ values were calculated in GraphPad Prism using four-parameter logarithmic regressions with upper and lower bounds of 100% and 0% neutralization, respectively. These IC_50_ values were then used to generate the heat map, using 0 and the highest IC_50_ value of a MAb against its own escape virus (KL-HAP-6B12 against VSV-ANDV^6B12^; 346.7 μg/ml) as the upper bound. Nonconvergent regressions were noted as having an infinitely large IC_50_ value. Download FIG S4, DOCX file, 0.2 MB. Copyright © 2020 Duehr et al. 2020 Duehr et al. This content is distributed under the terms of the Creative Commons Attribution 4.0 International license.

10.1128/mBio.00028-20.5 FIG S5 Cross-neutralization FRNAs of VSV-ANDV escape mutants. Clonal populations of each escape mutant virus in the presence of 128 IC_50_ of MAb were isolated via plaque picking from infected Vero cells. A stock of three clones of each virus was then grown, and FRNAs (and sequencing) were performed on these clones. Each graph (A to M) represents two experiments conducted on a single clone of the indicated escape mutant virus, with two technical replicates. All trend lines are logarithmic regressions except where such regressions were not converged; in these cases, connecting lines were used instead. Due to time and material constraints, FRNAs were not repeated with lower concentrations as described for Fig. 3. The negative-control antibody in each case was consistent across the entire escape virus but may vary between graphs. Each experiment used either KL-2G12 or KL-1D7 as negative control. Download FIG S5, DOCX file, 1.2 MB. Copyright © 2020 Duehr et al. 2020 Duehr et al. This content is distributed under the terms of the Creative Commons Attribution 4.0 International license.

### Protection studies in the Syrian hamster model of ANDV infection.

**FIG 6:**
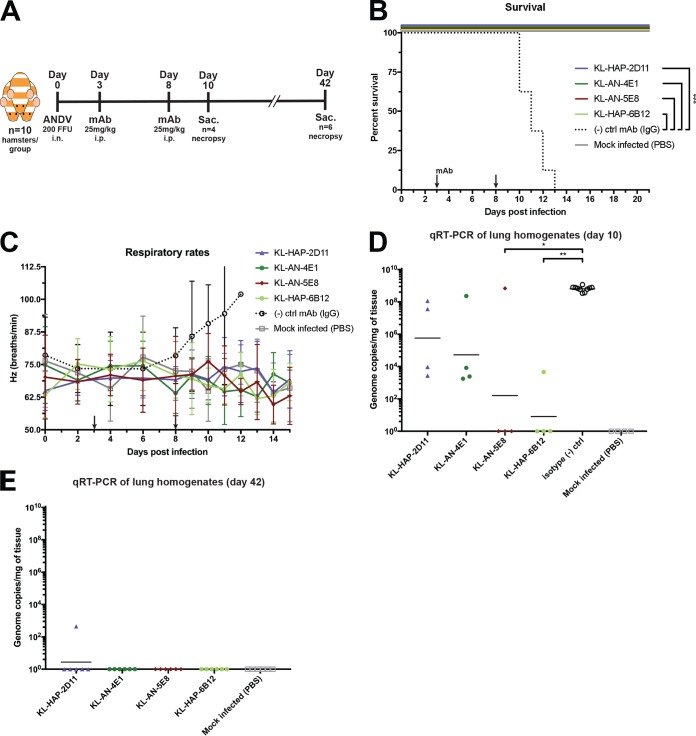
*In vivo* studies in the Syrian hamster model of HCPS. (A) Syrian hamsters were inoculated intranasally (i.n.) with 200 FFU of ANDV^CHI-9717869^ or PBS (mock) and then injected i.p. with 25 mg/kg of MAb or PBS on day 3 and day 8 postinfection. Experimental overview, including necropsy endpoints. (B) Survival of the indicated treatment groups shown to day 21 (though all experimental groups survived until the predetermined survival endpoint of day 42). ***, *P* < 0.0001 via Mantel-Cox log-rank test. (C) Respiratory rates of each hamster depicted as an average/group. (D) Viral genome copies per milligram of homogenized lung tissue on day 10 postinfection. *, *P* = 0.0161; **, *P* = 0.0048 via Kruskal-Wallis test incorporating Dunn’s test for multiple comparisons. (E) Same as for panel D but on day 42 postinfection.

The MAbs evaluated here have a variety of different properties, epitopes, and binding affinities. To evaluate their potential as postexposure therapeutics, we utilized the Syrian hamster model of ANDV infection and tested a subset of the mAbs for protective efficacy. This model has a broad similarity to HCPS in humans, including enhanced respiratory distress, high lethality, and select tissue tropisms (24, 50, 51). We employed a subset of neutralizing MAbs which span the properties and epitopes explored above, namely, KL-HAP-2D11 (Gc binding, ADCC inactive), KL-AN-4E1 (Gn binding, ADCC active), KL-AN-5E8 (Gn binding, ADCC active), and KL-HAP-6B12 (Gc binding, ADCC active). In the postexposure therapeutic setting (25 mg/kg of body weight each given on days 3 and 8 postinfection), the administration of all four MAbs resulted in 100% survival (Fig. 6A) (*n *= 6 per group, monitored up to day 42 postinfection). Hamsters given an equivalent dose of IgG control MAb (KL-2G12) exhibited 0% survival in comparison, succumbing to infection on days 10 to 13 postinfection (*P* = 0.0003 via Mantel-Cox log rank test). An important sign of morbidity in ANDV-infected hamsters is an increased respiratory rate, since animals do not appreciably lose weight or develop other clinical signs (51). When assessing respiratory rate, only animals in the IgG control-treated group displayed increased respiration before succumbing to infection within 1 to 2 days (Fig. 6B and C). We were also interested in viral replication in the lungs as a proxy for *in vivo* neutralization. On day 10, ANDV genome was still present in the lungs of most hamsters given KL-HAP-2D11 and KL-AN-4E1, but a significant reduction in titers occurred in hamsters treated with KL-AN-5E8 and KL-HAP-6B12 compared to that in control animals (Fig. 6D) (*n *= 4; *P* < 0.0001 via one-way analysis of variance [ANOVA]). By day 42, all animals in all experimental groups (with the exception of KL-HAP-2D11) had no detectable viral RNA in the lungs (Fig. 6E).

## DISCUSSION

As current environmental and economic trends continue, including climate change, deforestation, and habitat destruction, it is likely that orthohantavirus outbreaks will only increase in intensity, reach, and frequency. The recent ANDV outbreak in Epuyén in Argentina’s Chubut province demonstrates the public health concern posed by hantavirus infections. By late January 2019, there were at least 60 suspected cases and 11 known deaths, and human-to-human transmission was suspected. Public safety concerns led to large-scale quarantine in Epuyén (6). Despite the threat that New World orthohantaviruses, especially ANDV, might pose to the public health and safety of several nations, no effective vaccines, antiviral drugs, or immunological therapies have been approved for treatment or prevention. However, passive transfer of serum from human survivors and vaccinated animals has proved effective in pre- and postexposure treatment of infected Syrian hamsters (52–54). While this is useful for knowledge of the mechanisms of protection, polyclonal serum is expensive to produce and has many more associated risks (55). Recombinantly produced MAbs, on the other hand, are proving more promising every year as a drug monotherapy and cocktail approach to many diseases, including respiratory syncytial virus, Zaire ebolavirus, and human immunodeficiency virus 1 (HIV-1) (56–62). Here, we report the development and characterization of MAbs against the ANDV GnGc glycoprotein spike complex that display a wide variety of effective properties as potential monovalent or polyvalent therapies against HCPS (Table 1). These properties include neutralization, effector functions, and distinct binding sites. We identified 12 neutralizing antibodies against VSV-ANDV and authentic ANDV and eight ADCC-active MAbs.

**TABLE 1:** MAb characteristics (neutralization, affinity, effector functions, and epitopes)

MAb	Mouse isotype	ELISA (AUC)			Neutralization (IC_50_)		Effector (ADCC [AUC])	Escape mutation(s) (VSV-ANDV)
		ANDV rGn	VSV-ANDV	VSV-WT	VSV-ANDV	Auth. ANDV		
KL-AN-1F12	IgG2a	0.036	2.171	0.080	0.477	0.673	92.68	A871Q
KL-AN-3F6	IgG2b	5.457	4.078	0.095	2.049	1.085	144.90	D121V
KL-AN-4B6	IgG2a	0.013	0.054	0.096	∞	ND^*a*^	4.66	
KL-AN-4E1	IgG2a	4.129	3.051	0.101	1.013	0.843	65.18	N108K
KL-AN-4G11	IgG2b	5.480	3.420	0.049	1.190	1.433	92.54	K124N
KL-AN-5B3	IgG2a	0.022	0.052	0.030	397.200	ND	2.52	
KL-AN-5E8	IgG2b	5.597	2.958	0.028	0.353	1.107	95.72	D121G
KL-AN-5F1	IgG2b	0.022	0.039	0.029	∞	ND	4.12	
KL-AN-5F7	IgG2a	0.024	0.084	0.023	92.780	ND	3.72	
KL-AN-6B2	IgG2b	0.057	2.283	0.055	∞	ND	3.61	
KL-HAP-1D4	IgG2a	0.015	0.049	0.080	1,035	ND	8.30	
KL-HAP-1D8	IgG2a	0.015	0.059	0.080	∞	ND	11.65	
KL-HAP-2D11	IgG1	0.019	2.132	0.040	2.782	1.325	8.46	S831Y
KL-HAP-2F7	IgG1	0.022	2.296	0.044	3.768	2.303	6.91	P830S
KL-HAP-4E5	IgG2a	0.018	2.297	0.085	0.543	3.507	24.07	N939H
KL-HAP-4H6	IgG2a	1.237	2.575	0.030	8.484	0.251	13.69	K225R
KL-HAP-5G4	IgG2a	0.026	2.294	0.020	2.420	5.355	63.84	S883R; N939D; C1129F
KL-HAP-5H9	IgG2a	0.034	2.154	0.066	2.073	4.692	98.65	N939D; C1129F
KL-HAP-6B12	IgG2a	0.054	1.713	0.248	3.001	20.970	54.30	S883I; N939D
Positive control	IgG2a	3.079	6.782	7.262	ND	ND	66.45	
Negative control	IgG2a	0.027	0.056	0.031	∞	0.000		

a ND, not determined.

Another interesting aspect of our findings is the location of putative epitopes from escape mutants that were visualized on computational models of the ANDV Gn and Gc (pre- and postfusion). Taken together, the location of these epitopes and the properties of the corresponding MAbs may indicate a specific structural relationship between the GnGc and the membrane. If our models and computational fit are accurate, it would suggest that the ANDV Gn has an immunodominant role compared to that of the Gc. The ANDV Gn is likely membrane distal, and Gn epitopes are some of the most common and immune stimulatory patterns on the envelopes of ANDV and Sin Nombre virus (SNV) (53, 63). This is consistent with our data and the structural accounts of other orthohantaviruses, including TULV, PUUV, and HTNV (11, 43). This relationship is also reminiscent of many viral glycoprotein complexes, including influenza virus (hemagglutinin head versus stalk, hemagglutinin versus neuraminidase), HIV-1 (V2 epitope of gp120 versus membrane proximal epitopes), and tick-borne encephalitis virus (domains A versus B on E2) (43, 64–66).

Interestingly, the homologous vaccine regimen produced a majority of Gn-reactive MAbs, while the inverse (mostly Gc reactive) was true for the heterologous regimen. This could be the result of differences in amino acid conservation between these two proteins, given that the Gn is very diverse among orthohantaviruses and the Gc is relatively conserved (Fig. 1). This especially makes sense in light of recent data showing that heterologous prime-boost regimens involving different antigens from within a taxonomical family are effective at inducing responses to conserved and immunosubdominant glycoprotein epitopes (31, 34). A major caveat, though, is that this is an examination of a limited number of antibodies from only one mouse each from these two vaccination groups, and so our findings could be the result of stochasticity in a small number of hybridoma fusions.

These 12 neutralizing antibodies appear to bind to 7 distinct epitopes, increasing their utility in any cocktail therapies. Some attention was paid to the best possible setting for testing these MAbs against ANDV disease *in vivo*. As the Syrian hamster model does not demonstrate a pronounced symptomatic course but instead respiratory distress quickly followed by mortality, postexposure presymptomatic administration was chosen to demonstrate the possible utility of these MAbs in an outbreak scenario. When evaluated as monotherapies, KL-HAP-2D11, KL-AN-4E1, KL-AN-5E8, and KL-HAP-6B12 all provided 100% protection against ANDV-induced disease in Syrian hamsters. On day 10 postinfection, several animals in each experimentally treated group still had detectable viral genome in the lungs, though whether or not this corresponds to replicative virus is unknown. By day 42, all but one animal (treated with KL-HAP-2D11) had no detectable viral genome in lung homogenates. The dose used, 25 mg/kg, translates to approximately 125 μg/ml of serum in hamsters *in vivo* according to the literature (67). This is a value well above the neutralizing concentration of the isolated antibodies and a dose that might be feasible in humans as well, since, e.g., antibody therapeutics for ebolavirus infection have been given up to doses of 150 mg/kg (68). However, much lower concentrations than 25 mg/kg might be effective as well. Given the emergence of escape mutants in other *in vitro* (69) and *in vivo* studies (54, 70), it is remarkable that our antibodies protected very robustly as monotherapies. This might be due to the high dose administered or, potentially, to the epitopes targeted. The possibility of *in vivo* escape mutants remains, though it appears to have little bearing on animal survival.

In the recent study published by Garrido et al. (24), recombinant human MAbs were successfully evaluated for efficacy in a cocktail setting in the same Syrian hamster model of ANDV. In future studies, a similar cocktail approach could be explored with our MAbs to perhaps abrogate any detectable viral RNA in the animals posttreatment. However, it has to be noted that detectible viral RNA might not necessarily indicate infectious virus. By taking this into account, the monotherapy treatment with at least two of the MAbs seemed to have a strong impact on clearance in addition to protection from morbidity and mortality. Likewise, a combination of different characteristics (binding target, isotype, neutralization versus ADCC, etc.) could be employed to enhance the efficacy and longevity of any therapeutic regimen. Targeting several epitopes simultaneously, across Gn and Gc, may prove most effective and also most resistant to any resistance mutations. Studies conducted with other hemorrhagic fever viruses, including Ebola and Lassa viruses, have demonstrated the clear advantages of cocktail MAb approaches (71). Despite these unanswered questions, the data reported here may aid the future development of vaccines, therapeutics, and diagnostic tools against ANDV and other orthohantaviruses.

## MATERIALS AND METHODS

### Phylogeny and conservation map.

The neighbor-joining phylogenetic tree shown in Fig. 1A was constructed in FigTree v1.4.3 using proximity data from a multiple-sequence alignment (MSA) of amino acid sequences from the M segments of each of the viruses, listed in Table S1 in the supplemental material. The MSA was built in Clustal Omega v1.2.4 (72, 73); esthetic changes and clade categories for the tree were added in Adobe Illustrator. The conservation map in Fig. 1B was constructed from a separate MSA generated from GnGc amino acid sequences of the viruses listed on the left side of the figure. Black color indicates less conserved and white color indicates more-conserved residues. These viruses were selected as a representative sample from each of the three pathogenic hantavirus clades. Conservation scores at each amino acid position were obtained using AACon via JalView. These scores were then visualized on a gradient of black to white in Microsoft Excel. All notations on the diagram of GnGc are made to-scale based on notations from UniProt entry Q9E006 (74).

### Virus and cell culture.

Vero.E6 cells were obtained from the American Type Culture Collection (ATCC CRL-1586) and maintained in Dulbecco’s modified Eagle’s medium (DMEM; Gibco), made complete with 10% fetal bovine serum (FBS; Clontech), 1% 1 M 4-(2-hydroxyethyl)-1-piperazine ethanesulfonic acid (HEPES; Gibco), 100 μg/ml of streptomycin, and 100 U/ml of penicillin (working concentration, mixture sold as PenStrep; Gibco). Sf9 insect cells (ATCC CRL-1711) were propagated in *Trichoplusia ni* medium-Fred Hinks (TNM-FH; Gemini Bio-Products) made complete with 10% FBS and PenStrep antibiotic mixture. High Five cells (BTI-TN-5B1-4 subclone; Vienna Institute of Biotechnology) (75) were grown in serum-free SFX medium (HyClone) made complete with PenStrep antibiotic mixture.

VSV-ANDV (originally described as VSVΔG/ANDVGPC [28]) is a replication-competent recombinant virus rescued on the background of vesicular stomatitis virus (VSV). In this case, the coding region for VSV-G has been excised from pVSV-XN2, a plasmid transcribing the positive-sense complement to the VSV genome (Indiana strain) used in the rescue of VSV (76), and replaced with the coding region from the M segment of Chilean ANDV strain 9717869 (28, 74). VSV expressing a wild-type G (VSV-WT) was also used. VSV-ANDV and VSV-WT were propagated and grown in Vero.E6 cells in minimal essential medium (MEM) made complete with 1% HEPES (1 M), 1% PenStrep, 1% l-glutamine (200 mM; Gibco), 1.6% sodium bicarbonate (Gibco), and 0.6% bovine serum albumin (BSA). After 3 days incubation at 37°C, viral supernatants were sterile filtered via 0.22-μM membrane (EMD Millipore) and aliquoted for storage at −80°C. Viral stock titers were determined in Vero.E6 cells via a focus-forming unit assay as described previously and stained with MAb KL-AN-4E1 (VSV-ANDV) or monoclonal anti-VSV-N (clone 10G4; Kerafast), both diluted 1:1,000 in blocking buffer (30). Foci were then counted and titers calculated using Microsoft Excel.

Authentic Andes orthohantavirus (strain HI-9717869) was propagated as follows: In brief, 1.5 × 10^5^ plaque-forming units (PFU) of ANDV was added dropwise onto a confluent monolayer of low-passage-number Vero.E6 cells in a 6-well plate after washing thoroughly with phosphate-buffered saline (PBS) (pH 7.4; Gibco). Cells were then maintained in MEM at 37°C during incubation with 5% CO_2_. Every 3 days, cells were trypsinized and expanded. The first passage was from a 6-well plate into a T75 flask and then from a T75 flask into a T175 flask (with complete DMEM [cDMEM] for each subsequent passage). Passaging then proceeded from one T175 into three T175s and finally from three T175s into nine T175s. These nine flasks were then incubated at 37°C for 7 days. On the fourth day, a bolus of 60 ml medium was added to each flask. The supernatant from these flasks was then combined and sterile filtered via a 0.22-μM membrane (EMD Millipore). This sterile filtrate was then concentrated 1:120 using 100 kDa Amicon centrifugation filters (EMD Millipore) spun at 4,000 rpm at 4°C. Membranes were then washed 3× with PBS to remove any additional medium products. The resulting concentrate was then aliquoted and frozen to −80°C, and the titer was determined as described above. For each authentic hantavirus, an anti-N polyclonal IgG produced in rabbit was used as primary stain (1:1,000, NR-9673; BEI), and an anti-rabbit IgG linked to horseradish peroxidase was used as secondary stain (1:1,000; GE Healthcare). All work with authentic hantaviruses was performed in a CDC-inspected biosafety level 3 (BSL3) laboratory.

### Vaccination and hybridoma fusion.

The overall vaccination schema and hybridoma screening process is summarized in Fig. S1. All animal procedures were performed in accordance with the Icahn School of Medicine at Mount Sinai Institutional Animal Care and Use Committee (IACUC). DNA vaccination and hybridoma fusion were performed as described in detail previously (30). Female BALB/c mice (6- to-8-weeks-old; sourced from Jackson Laboratory) were vaccinated by intramuscular injection of 100 μg DNA preparations suspended in 50 μl of water for injection (WFI), followed by electrical stimulation (TriGrid delivery; Ichor Medical Systems) (77). The TriGrid electrode array is spaced in 2.5-mm intervals. The field has an amplitude of 250 V/cm of electrode spacing. Six pulses are provided for a 40-ms duration applied in 400-ms intervals. A homologous group (AN) was given two vaccinations of ANDV M DNA in pCAGGS and then an intraperitoneal (i.p.) injection of 10^5^ PFU VSV-ANDV for the third vaccination. A heterologous-prime group (HAP) was vaccinated with HTNV M DNA, followed by ANDV M DNA, followed by PUUV M DNA. In both groups, each vaccination was separated by 4 weeks in order to allow for a full immune response to occur and be integrated into B cell memory (29, 30). Four weeks after the last vaccination, one mouse from each group was given 10^5^ PFU VSV-ANDV via i.p. injection. Three days later, both mice were sacrificed, and their spleens were excised in a laminar flow hood for use in hybridoma fusions.

In this procedure, the spleen was homogenized by hand using toothless flat surgical tweezers to acquire a monocellular suspension of splenocytes. Splenocytes and Sp2/0 myeloma cells (in an exponential phase of growth) were washed three times and combined in a ratio of 5:1. Cell fusion was performed via dropwise addition of 1 ml polyethylene glycol (mass ∼4 kDa per polymer). The mixture was then resuspended in cDMEM and incubated overnight at 37°C. The next day, cells were centrifuged and resuspended in 10 ml cDMEM. This cell suspension was then combined with 90 ml semisolid ClonaCell-HY medium D (Stemcell Technologies) and laid out on 10 × 10-ml tissue culture dishes. Ten days later, colonies were picked individually and resuspended each in a single well of a 96-well plate filled with 100 μl ClonaCell-HY medium E. Two days later, 50 μl medium E was added to expand the culture for screening.

### Screening hybridoma clones.

Five days after picking and isolating clones, hybridoma supernatants were screened via immunostaining. Ninety-six-well plates were infected with either VSV-ANDV or VSV-WT and fixed with 100% ice-cold methanol overnight at 4°C. After blocking with 180 μl 3% nonfat milk for 1 h at room temperature (RT), plates were decanted and 50 μl of each clone was added to wells of both VSV-ANDV- and VSV-WT-infected plates and incubated for 1 h at RT. After washing three times with PBS, a secondary stain consisting of 1:1,000 anti-mouse horseradish peroxidase (HRP)-conjugated secondary antibody was added. Following a 1-h incubation at RT, plates were washed three times with PBS and then developed. For enzyme-linked immunosorbent assay (ELISA)-developed plates, SigmaFast *o*-phenylenediamine dihydrochloride (OPD) was added. After 10 min of incubation on the plates, the development reaction was stopped with 3 M HCl. The plates were then read for optical density at 490 nm (OD_490_), and a cutoff was devised for an optimal number of positive clones. In this case, a positive clone was a well which had high signal above background in VSV-ANDV-infected plates and at or below background in VSV-WT-infected plates. For plates developed via TrueBlue peroxidase substrate (SeraCare), reagent was added after the final wash was decanted, and the plates allowed to develop for 1 h at RT. Three micrograph images were then captured at ×20 magnification of every well under bright field. Positive versus negative determinations were made by eye based upon density of blue staining on the cell monolayer in both VSV-ANDV- and VSV-WT-infected plates. Positive clones were then isotyped using a Pierce ELISA-based rapid antibody isotyping kit (Life Technologies). Only IgG clones were expanded.

### Antibody purification.

Purification of MAbs was conducted as described previously (30, 34). Briefly, positive hybridoma clones were expanded and, when at approximately 12 million cells per culture, ∼8 million cells were frozen in a 1-ml cryostock each. Remaining cells were slowly expanded to large culture volumes (∼800 ml each) in hybridoma serum-free medium (Gibco), from which supernatants were harvested via low-speed centrifugation and sterile filtered via 0.22-μM membranes (EMD Millipore). These supernatants were then affinity purified via gravity flow with protein G-linked Sepharose 4 fast flow beads packed into columns (GE Healthcare). After washing the beads with 3 column volumes (∼500 ml) of sterile PBS (pH 7.4), an elution step was carried out with 45 ml of 0.1 M glycine-HCl buffer (pH 2.7). The eluate was then immediately neutralized with 5 ml of 2 M Tris-HCl buffer (pH 10) to bring the overall solution to a pH of approximately 7.0. The MAbs were then buffer exchanged to PBS (pH 7.4) using 30 kDa Amicon centrifugation filters (EMD Millipore) and washed three times with PBS on the membrane. Finally, the concentration of antibody was quantified using a Nanodrop spectrophotometer (Thermo Scientific), measuring absorbance using the protein *A*_280_ protocol.

### Generation of recombinant proteins via baculovirus expression.

The coding sequence of the ANDV strain CHI-9717869 Gn ectodomain (as defined using transmembrane prediction software TMHMM v2.0 [78, 79]), starting with methionine and excluding the hydrophobic region after amino acid 450 (nucleotide [nt] 1,350), was amplified from a synthesized and codon-optimized gene using primers that contained the coding sequence for a hexahistidine tag on the carboxy terminus and BamHI and NotI restriction sites on the amino and carboxy termini, respectively. The PCR product was cut using HF versions of the specified enzymes (New England BioLabs [NEB]) and then ligated into a modified pFastBacDual vector in front of the polyhedrin promoter using T4 ligase (NEB). The constructs obtained this way were then sequence confirmed via Sanger sequencing and transformed into DH10Bac competent cells (Thermo Fisher Scientific). The transformed bacteria were grown on LB agar plates containing X-Gal (5-bromo-4-chloro-3-indolyl-β-d-galactopyranoside), tetracycline, kanamycin, and gentamicin (Gibco) in order to produce recombinant bacmid via blue-white screening as described previously (30, 36, 80). This bacmid was then transfected into Sf9 cells, in order to rescue recombinant baculovirus, which was propagated in Sf9 cells, and finally used to infect High Five cells, in order to more efficiently produce recombinant protein. Recombinant Gn was then purified from the supernatants of these High Five cell cultures using gravity flow and Ni^2+^-nitrilotriacetic acid (NTA) beads (Invitrogen) according to a published protocol (36). The resulting protein preparation was aliquoted and frozen at −80°C. A thawed aliquot was measured to determine protein concentration via the Bradford assay.

### Virus purification via ultracentrifugation on a sucrose gradient.

VSV-ANDV and VSV-WT were purified using a protocol originally described for virus-like particles (30). In brief, virus was cultured in Vero.E6 cells as described above. Supernatants were sterile filtered and added slowly to a 30% sucrose cushion in NTE buffer (0.5 M NaCl, 10 mM Tris-HCl [pH 7.5], 1 mM EDTA), to avoid disrupting the virus-cushion interface. This layered column was centrifuged at 28,000 rpm for 2 h at 4°C in a Beckman L7 ultracentrifuge with SW-28 rotor (75). The pellet was resuspended in PBS, aliquoted, and frozen at −80°C. The titer was determined from a thawed aliquot via a focus-forming unit assay, and protein concentration was measured using the Bradford assay.

### ELISAs.

ELISAs were performed as described previously (30, 81–83). In brief, antigen was added to Immulon 4 HBX plates overnight at 4°C at a concentration of 2 μg/ml (protein) or 5 μg/ml (purified virus) (50 μl/well) in coating buffer (KPL, pH 7.4; SeraCare). After firmly decanting coating buffer, 180 μl/well of blocking buffer (3% nonfat milk in PBS [pH 7.4] containing 0.1% Tween 20 [PBS-T]) was added to the plates. After incubating for 1 h at RT, primary stain diluted in blocking buffer was added to columns 2 to 11 lengthwise (typically, 30 μg/ml initially with a 1:3 serial dilution). Blocking buffer only was added to columns 1 and 12 and rows A and H for background calculation purposes. After 1 h in a 20°C incubator, plates were washed four times with PBS (pH 7.4) containing 0.1% Tween 20 (PBS-T). Secondary stain (anti-mouse polyclonal IgG conjugated to HRP [Rockland]; 1:1,000 in blocking buffer) was then added to every well, and the plates incubated for 1 h in a 20°C incubator. Plates were then washed four times with PBS-T and developed. Initially, 100 μl/well of SigmaFast OPD was added. After 10 min of incubation on the plates, the development reaction was stopped with 50 μl/well of 3 M HCl. The plates were then read at an optical density of 490 nm (OD_490_), and background calculation and removal were performed in Microsoft Excel. The data were plotted in GraphPad Prism, and best-fit curves were determined using 4-parameter logarithmic regression with aforementioned background values.

### Immunostaining of infected cells.

Immunostaining was conducted similarly to the ELISA method described above. Briefly, Vero.E6 cells were infected with a given virus at a multiplicity of infection (MOI) of either 0.5 or 1.0, incubated for a set duration (optimized for each virus used), and then fixed with either 3.7% formalin or 100% ice-cold methanol (typically, the latter was used for anti-internal protein positive-control MAbs) overnight at 4°C. Then, fixation buffer was removed via vacuum aspiration and blocking buffer was added. After incubating for 1 h at RT, blocking buffer was removed and primary stain added diluted in blocking buffer at the designated concentration. After 1 h incubation at RT, plates were washed three times with PBS, and then secondary antibody (either anti-mouse HRP or anti-rabbit HRP conjugate depending on the primary stain) was added 1:1,000 in blocking buffer to the plates. After 1 h incubating at RT, plates were washed three times with PBS. TrueBlue peroxidase substrate was then added, and the plates incubated for 1 h at RT before images were captured using an EVOS microscope at ×20 magnification under bright field. All work with authentic hantaviruses was performed in a CDC-inspected BSL3 laboratory.

### Western blots.

Binding of MAbs to the antigen of interest was assessed using sodium dodecyl sulfate-polyacrylamide gel electrophoresis (SDS-PAGE; 2% to 10% polyacrylamide gradient) (Mini Protean TGX gels; Bio-Rad) under reducing and nonreducing conditions. Vero.E6 cells were infected with a multiplicity of infection (MOI) of 1 of either VSV-ANDV or influenza virus (A/Netherlands/602/2009 [H1N1]) and then harvested after 48 h of incubation at 37°C. Ten microliters of a 2-ml suspension of ∼8 × 10^6^ Vero.E6 cells was then loaded onto a 2% to 10% gradient SDS-PAGE gel and electrophoresed at 200 V for 40 min After electrophoresis, each gel was transferred to a polyvinylidene difluoride (PVDF) membrane via either Owl HEP series semidry or iBlot 2 dry electroblotting systems (Thermo Scientific) according to the manufacturer’s instructions. The membranes were then washed once with PBS-T and blocked with 3% nonfat milk in PBS-T (blocking buffer) for 2 h with shaking at RT. The membranes were then washed three times with PBS-T, and primary stain added as indicated above, diluted in blocking buffer. After incubation with the membrane for 1 h shaking at RT, the primary stain was removed, and the membrane was washed three times with PBS-T. A secondary stain composed of anti-mouse IgG linked to alkaline phosphatase (AP) was then diluted 1:1,000 in blocking buffer and incubated with the membrane for 1 h with shaking at RT. Then, the membrane was washed three times with PBS-T and developed using an alkaline phosphatase conjugate substrate kit (1706432; Bio-Rad).

### FRNAs and focus-forming unit assays.

FRNAs were conducted using a modified version of a protocol established previously (84). In brief, MAbs were serially diluted 1:5 in infectious medium (MEM supplemented with 0.21% BSA [Fischer Scientific], 100 μM HEPES [Gibco], PenStrep, 20 mM l-glutamine [Gibco], and 0.37% [3.7 g/liter] sodium bicarbonate [Gibco]), with a starting concentration of 120 μg/ml. The negative-control antibody was one of two murine IgG2a antibodies, KL-2G12 or KL-1D7, specific for *Zaire ebolavirus* or *Influenza A virus*, respectively (30). These MAb dilutions were then incubated with ∼70 PFU of the indicated virus (VSV-ANDV, escape mutants thereof, or authentic ANDV) for 1 h shaking gently in a 25°C incubator. These mixtures were then incubated on 80% to 100% confluent monolayers of Vero.E6 cells in 12-well plates for 1 h at 37°C, shaking every 15 min. The cells were overlaid with 0.64% agarose (Oxoid) in MEM containing approximately the same concentration of MAb as the inoculum. After 3 days (for VSV-ANDV or escape mutants thereof) or 4 days (for authentic ANDV) of incubation at 37°C, 3.7% formalin was added as a fixative. After fixation overnight at 4°C, agar and formalin were removed, and the cells were immunostained as described above with KL-AN-4E1 or KL-AN-1F12 (KL-AN-4E1 for all MAb dilutions except KL-AN-4E1, which was stained with KL-AN-1F12 to circumvent competitive inhibition). Primary dilutions of the MAbs were 1:1,000. Development was performed using TrueBlue peroxidase substrate (SeraCare). Individual foci were counted in each well, and percent inhibition was calculated in Microsoft Excel, comparing values to wells containing no MAb. All assays were performed in duplicates. The data were analyzed using GraphPad Prism, and the concentration at which a given MAb inhibited 50% of focus formation (IC_50_) was calculated using a nonlinear 4-parameter logarithmic regression.

Focus-forming unit assays were performed similarly to FRNAs, except virus stocks were serially diluted 1:10 in MEM without any MAb across 6 wells of a 24-well plate. These dilutions (inoculum) were then added directly onto 80% to 100% confluent monolayers of Vero.E6 cells after washing with PBS to remove any latent serum. After 1 h of incubation at 37°C, agitating every 15 min, inoculum was removed, and MEM containing 0.64% agarose and 0.001% DEAE-dextran overlaid on top. Incubation and staining then proceeded as described above. Foci were then counted per well, and the titer was calculated in Microsoft Excel. All work with authentic hantaviruses was performed in a CDC-inspected BSL3 laboratory.

### Escape mutant heatmap.

IC_50_ values were extracted from each FRNA graph in GraphPad Prism using four parameter logarithmic nonparametric regression, with upper and lower bounds set at 100% and 0% inhibition, respectively. Nonconverging regressions were given an infinitely large IC_50_. A heat map was then generated and stylized in Microsoft Excel, using the highest value for a MAb against its own escape virus (KL-AN-6B12 against VSV-ANDV^6B12^; 346.7 μg/ml) as the upper bound.

### Generation of escape mutations.

MAb escape mutant viruses were generated using VSV-ANDV via serial passaging in Vero.E6 cells in the presence of incrementally increasing amounts of MAb. The starting concentration was 2× IC_50_ (as calculated from FRNAs of each MAb against VSV-ANDV). To begin, Vero.E6 cells in 12-well tissue culture plates (Sigma) were infected with VSV-ANDV at an MOI of 1 with 2× IC_50_ of MAb (performed in duplicates) in MEM. After 72 h of incubation at 37°C, 200 μl of supernatant was collected from each culture and used to directly inoculate a fresh monolayer of Vero cells in the presence of a 2-fold increase in the MAb concentration. The remaining supernatant was aliquoted and frozen at −80°C. The monolayer was then fixed and immunostained as described elsewhere in these methods to confirm the presence of infectious virus. If a passage did not stain properly, a prior aliquot with positive staining was thawed and passaging resumed. This process was repeated until each MAb was at 128× IC_50_ (a total of 6 passages) with virus presence confirmed by immunostain. Virus was additionally passaged in the presence of an irrelevant mouse MAb against the VSV-ZEBOV (KL-2G12) to control for variants that occur as a result of passaging alone. Escape mutant viruses were plaque purified after serial passaging to obtain monoclonal stocks. These monoclonal stocks were then tested in FRNAs against their corresponding escape MAbs with no residual neutralization activity detected. Sequencing of monoclonal stocks was then performed using the following primers to amplify the ANDV M insert in the VSV-ANDV genome: forward, 5′-ATGATGATGATGGAAGGGTGGTATCTGGTTG-3′; reverse, 5′-ATGATGATGTTAGACAGTTTTCTTGTGCCCTCTCC-3′. Three monoclonal isolates for each MAb were sequenced, and alignment was performed of each read to the VSV-ANDV genome.

### Antibody-dependent cellular cytotoxicity reporter assays.

ADCC capacity of each MAb was measured using an ADCC Reporter Bioassay kit (Promega) largely in line with the manufacturer’s instructions. In brief, 3 × 10^4^ Vero.E6 cells per well were seeded in white-bottom 96-well plates (Corning) and infected with VSV-ANDV in MEM at an MOI of 1 16 h later. After 2 days of incubation with virus at 37°C, 3-fold serial dilutions of each MAb were prepared in MEM, starting with 90 μg/ml. A negative-control antibody (KL-2G12) was used as well for determining the background. These MAbs were then added to the infected plate with effector cells from the Promega kit at a ratio of 3:2 (effector cells to infected cells) and additional medium such that the effective starting concentration of each MAb was 30 μg/ml. The plates were then incubated at 37°C for 6 h. Bio-Glo Luciferase reagent (Promega) was then added, and luminescence was measured immediately.

### Computational model and fit of ANDV Gn and Gc.

To generate a computational model of ANDV Gn, a pairwise sequence alignment was performed between the amino acid sequences of Tula virus (TULV) and ANDV Gn using EMBOSS-Needle (72, 85). This alignment was then used to perform a structural model of the ANDV amino acid sequence mapped onto a 16-Å resolution structure of the TULV Gn published by Li et al. (PDB 5FXU) (43). The model was constructed using SWISS-MODEL, relying on the 73.8% similarity and 56.4% identity of the two protein sequences in the relevant domains (86, 87). The model fidelity was verified using a structural alignment in UCSF Chimera. This modeled structure was then computationally fit inside the cryo-electron microscopic tomograph of the TULV envelope published by Li et al. (43) and used to hone the published TULV structure. This volumetric and stoichiometric fit was accomplished by segmenting the cryo-EM tomograph using the “segment map” and “fit to segments” tools included in Segger v1.9.5, set for 1,000 iterations (88). A predetermined correlation cutoff for each fit was set at >0.8. As a result, two nearly equivalent fits were observed (correlation, 0.8379 and 0.8530). The more well-correlated fit also corresponded to the orientation of the TULV Gn structural fit performed by Li et al. (43), and so this was used for visualization. Escape mutations present on the Gn model were visualized in Chimera 8.6.1 and PyMol v2.1.1.

To visualize escape mutations induced on the VSV-ANDV Gc, two separate computational models of ANDV Gc were constructed (one pre- and one postfusion; PDB identifiers 5LJY and 5LK3, respectively) based on two structures of the HTNV Gc published by Guardado-Calvo et al. (49). We then verified the fidelity and plausibility of these models using sequence and structural similarity. The sequences of ANDV Gc and HTNV Gc used for constructing these two models were 77.1% similar and 64.9% identical per EMBOSS-Needle. Structural fits performed in UCSF Chimera resulted in RMSD values of 0.082 and 0.121 for the pre- and postfusion Gc models, respectively. ANDV Gc escape mutations were then visualized in UCSF Chimera and PyMol. The postfusion ANDV Gc trimer was visualized by structurally aligning three monomers of the ANDV Gc model with each monomer of the HTNV Gc postfusion trimer (PDB 5LJY, biological assembly 1).

### Syrian hamster animal studies.

All work with ANDV-infected hamsters and potentially infectious animal material was conducted in a BSL4 laboratory at Rocky Mountain Laboratories (RML), Division of Intramural Research, National Institute of Allergy and Infectious Diseases, National Institutes of Health. Removal of any samples from high containment was only performed after inactivation via standard operating protocols approved by the RML Institutional Biosafety Committee. All animal experiments were approved by the RML Animal Care and Use Committee. All hamsters were group housed in HEPA-filtered cage units. All animal experiments were performed in compliance with the guidelines of the Association for Assessment and Accreditation of Laboratory Animal Care, International (AAALAC) by certified staff in an AAALAC-approved facility at RML. Animal procedures were carried out under isoflurane anesthesia by trained personnel, and all efforts were made to ameliorate animal welfare and minimize suffering. Food and water were available *ad libitum*, and the animals were monitored at least twice daily. Experiments were conducted in female hamsters 5 weeks of age or older (*n* = 10/group). Groups consisted of four experimental MAbs, one isotype IgG control MAb, and an uninfected untreated control group (6 total for a grand total of 60 hamsters). Each infected group received 200 focus-forming units (FFU) of ANDV (strain CHI-9717869) intranasally on day 0, followed by 25 mg/kg of the indicated MAb injected i.p. on both day 3 and day 8. Animals were evaluated for respiratory rate and survival every other day until any sign of illness was observed, at which point observations were taken every day. When the first animal of any group exhibited signs of morbidity (in this case, day 10 postinfection), 4 animals from each group were sacrificed and necropsied for lung viral titer estimation via reverse transcription-quantitative PCR (qRT-PCR). Surviving animals on day 42 postinfection were likewise necropsied.
