# Supplementary material for: Neutralizing Monoclonal Antibodies against the Gn and the Gc of the Andes Virus Glycoprotein Spike Complex Protect from Virus Challenge in a Preclinical Hamster Model
Source: mBio. 2020 Mar 24;11(2):e00028-20. doi: 10.1128/mBio.00028-20 (PMC7157512; doi:10.1128/mBio.00028-20)
Supplement: TABLE S1 [file mBio.00028-20-st001.docx]

**Suppl. Table 1.** Amino acid sequences used for the construction of Figure 1.

| **Tree Label** | **Virus Name** | **Strain/Isolate** | **Genbank Acc #** |
| --- | --- | --- | --- |
| ANDV | Andes virus | CHI-9717869 | [AY228238.1](https://www.ncbi.nlm.nih.gov/nuccore/AY228238.1) |
| DOBV | Dobrava-Belgrade virus | GRW/Aa | [JQ026205.1](https://www.ncbi.nlm.nih.gov/nuccore/JQ026205.1) |
| HTNV | Hantaan virus | 76-118 | [KT885048.1](https://www.ncbi.nlm.nih.gov/nuccore/KT885048.1) |
| MJNV | Imjin virus | Cixi-Cl-23 | [KJ420541.1](https://www.ncbi.nlm.nih.gov/nuccore/KJ420541) |
| PHV | Prospect Hill virus | M3 | [NC_038940.1](https://www.ncbi.nlm.nih.gov/nuccore/NC_038940.1) |
| PUUV | Puumala virus | Sotkamo | [NC_005223.1](https://www.ncbi.nlm.nih.gov/nuccore/NC_005223.1) |
| SEOV | Seoul virus | 80-39 | [NC_005237.1](https://www.ncbi.nlm.nih.gov/nuccore/NC_005237.1) |
| SNV | Sin Nombre virus | NM H10 | [NC_005215.1](https://www.ncbi.nlm.nih.gov/nuccore/NC_005215.1) |
| TPMV | Thottapalayam virus | VRC 66412 | [NC_010708.1](https://www.ncbi.nlm.nih.gov/nuccore/NC_010708.1) |
| TULV | Tula virus | Moravia/5302v/95 | [NC_005228.1](https://www.ncbi.nlm.nih.gov/nuccore/NC_005228.1) |
