# Supplementary material for: Neutralizing Monoclonal Antibodies against the Gn and the Gc of the Andes Virus Glycoprotein Spike Complex Protect from Virus Challenge in a Preclinical Hamster Model
Source: mBio. 2020 Mar 24;11(2):e00028-20. doi: 10.1128/mBio.00028-20 (PMC7157512; doi:10.1128/mBio.00028-20)
Supplement: FIG S1 [file mBio.00028-20-sf001.docx]

**
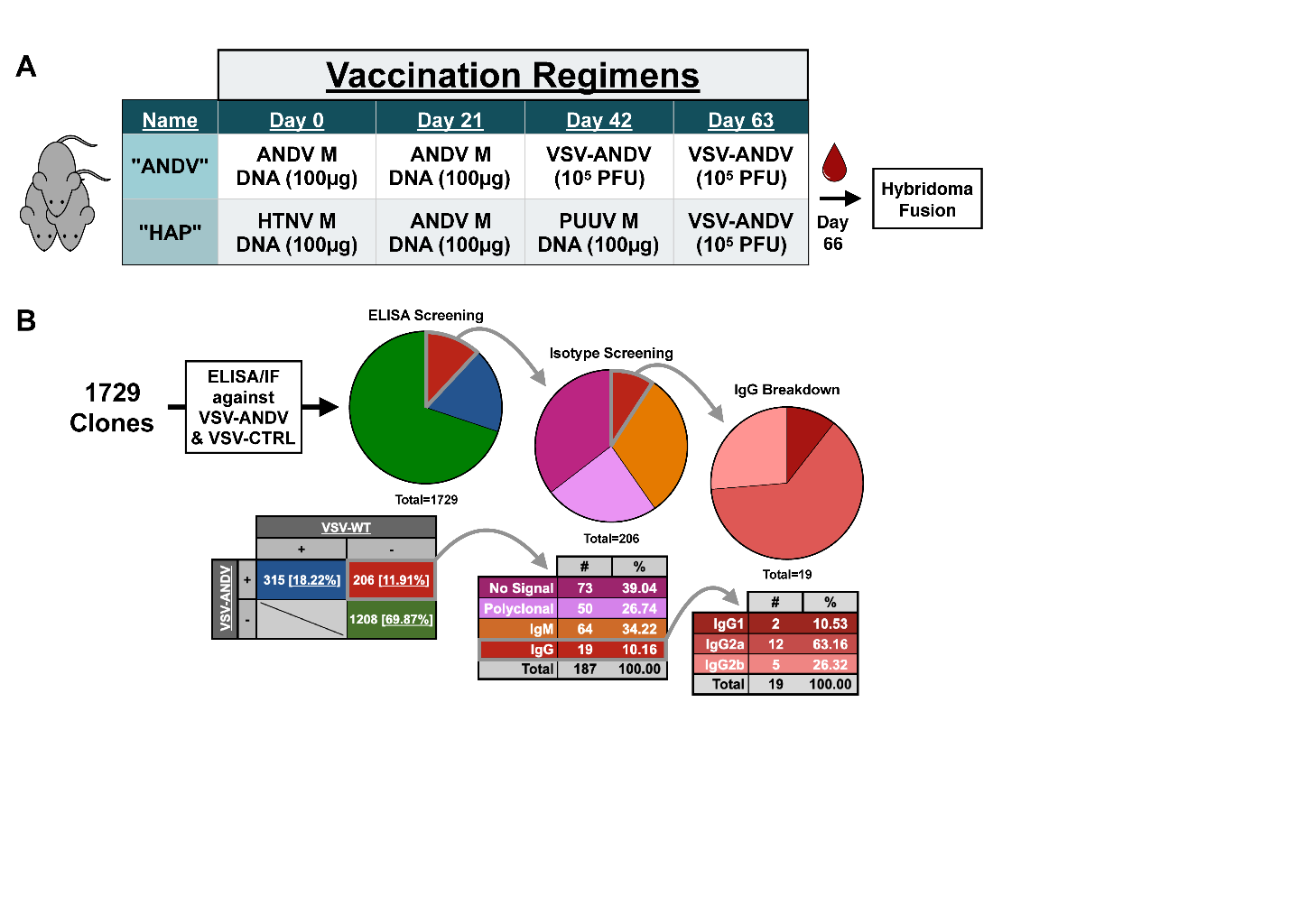
Suppl. Figure 1. Vaccination regimens and hybridoma screening statistics. A)** Two groups of five female BALB/c mice each were vaccinated with the given DNA encoded in pCAGGS mammalian expression plasmids in two or three separate events, 100 μg each, separated by 21-day intervals. DNA vaccinations were done via i.m. injection followed by electroporation at the injection site. Subsequent boost(s) were comprised of 10^5^ PFU VSV-ANDV injected i.p. Only the mouse used for hybridoma fusion was given the final boost. Three days after the last boost, the mouse was anesthetized and the spleen excised for use in a hybridoma fusion. **B)** Diagram showing the statistics of the ensuing two hybridoma fusions. Out of 1729 picked clones, 11.91% were positive for VSV-ANDV and negative for VSV-WT via ELISA and immunostaining of infected cells. Out of 206 clones in the aforementioned category (VSV-ANDV(+)/VSV-WT(-)), 19 were monoclonal IgG per rapid ELISA isotyping. Of those 19, two were IgG1, twelve IgG2a, and five IgG2b. These 19 clones were used for subsequent assays.
