# Supplementary material for: Neutralizing Monoclonal Antibodies against the Gn and the Gc of the Andes Virus Glycoprotein Spike Complex Protect from Virus Challenge in a Preclinical Hamster Model
Source: mBio. 2020 Mar 24;11(2):e00028-20. doi: 10.1128/mBio.00028-20 (PMC7157512; doi:10.1128/mBio.00028-20)
Supplement: FIG S2 [file mBio.00028-20-sf002.docx]

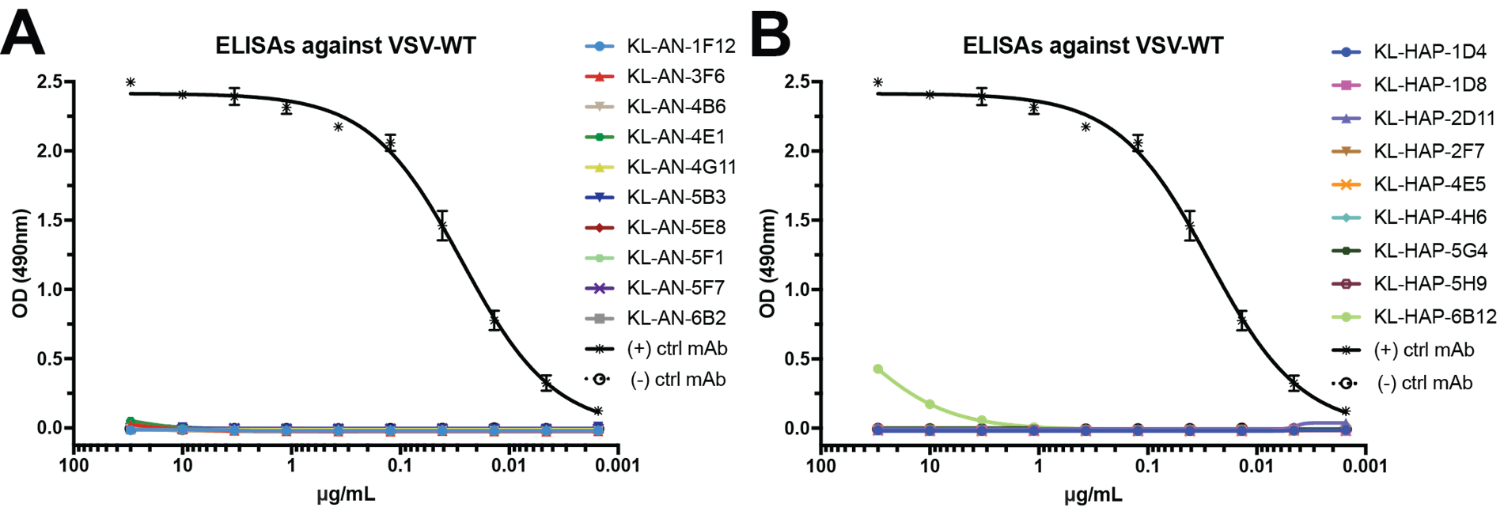


**Suppl. Figure 2. ELISAs against purified wildtype VSV. A)** and **B)** show ELISAs of mAbs from AN and HAP fusions (respectively) against VSV-WT. Experiments were conducted as in Figure 2A and C, with a shared positive and negative control.
