# Supplementary material for: Neutralizing Monoclonal Antibodies against the Gn and the Gc of the Andes Virus Glycoprotein Spike Complex Protect from Virus Challenge in a Preclinical Hamster Model
Source: mBio. 2020 Mar 24;11(2):e00028-20. doi: 10.1128/mBio.00028-20 (PMC7157512; doi:10.1128/mBio.00028-20)
Supplement: FIG S3 [file mBio.00028-20-sf003.docx]

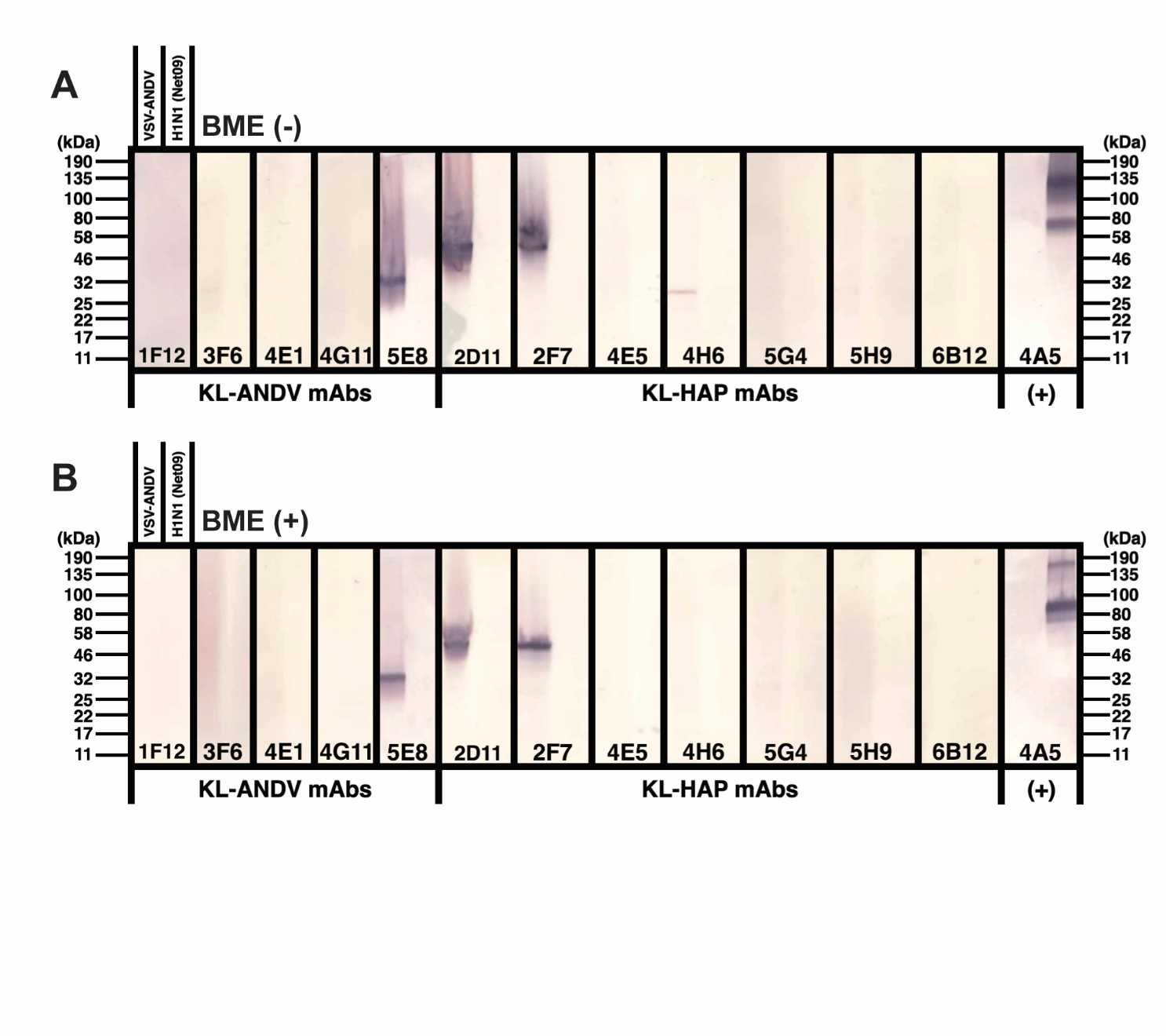


**Suppl. Figure 3. Western blots against lysates of cells infected with VSV-ANDV.** Vero.E6 cells were infected with an MOI of 1 of VSV-ANDV or influenza virus (A/Netherlands/602/2009 (H1N1)) and harvested after 48 hrs of incubation at 37°C. Cells were then resuspended in NP40 lysis buffer and combined 1:1 with Laemmli buffer either **A)** with or **B)** without BME (β-mercaptoethanol). Lysates were then run on a 2-10% gradient SDS-PAGE and blotted with each mAb at 30 μg/mL as primary stain. Secondary stain was anti-mouse alkaline phosphatase conjugated antibody (1:1000) and development was via AP development system (Bio-Rad). The ladder is based upon a color protein standard (Life Technologies).
