# Supplementary material for: Neutralizing Monoclonal Antibodies against the Gn and the Gc of the Andes Virus Glycoprotein Spike Complex Protect from Virus Challenge in a Preclinical Hamster Model
Source: mBio. 2020 Mar 24;11(2):e00028-20. doi: 10.1128/mBio.00028-20 (PMC7157512; doi:10.1128/mBio.00028-20)
Supplement: FIG S4 [file mBio.00028-20-sf004.docx]

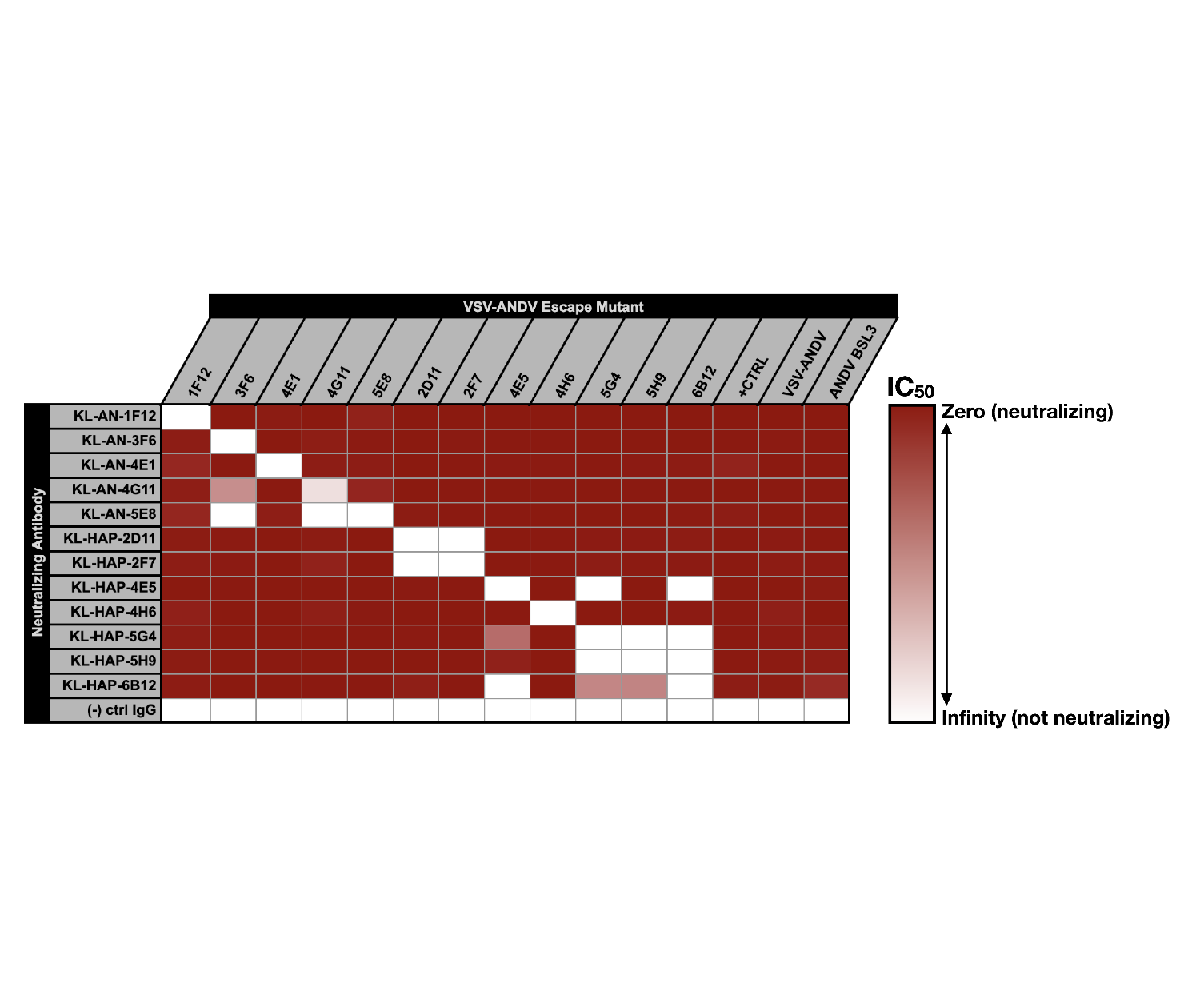


**Suppl. Figure 4. Heatmap of VSV-ANDV escape mutant IC_50_ values.** Escape mutants were plaque-purified to ensure monoclonal virus populations, before growth and titering. PRNAs of each neutralizing antibody against each escape virus were then conducted, to understand the relationship between epitopes and to confirm the escape phenotype. Full graphs of each PRNA are available in Suppl. Figure 6. IC_50_ values were calculated in GraphPad PRISM using four-parameter logarithmic regressions with upper and lower bounds of 100% and 0% neutralization, respectively. These IC_50_ values were then used to generate the above heatmap, using 0 and the highest IC_50_ value of a mAb against its own escape virus (KL-HAP-6B12 against VSV-ANDV^6B12^; 346.7 μg/mL) as the upper bound. Non-convergent regressions were noted as having an infinitely large IC_50_ value.
