# Supplementary material for: Neutralizing Monoclonal Antibodies against the Gn and the Gc of the Andes Virus Glycoprotein Spike Complex Protect from Virus Challenge in a Preclinical Hamster Model
Source: mBio. 2020 Mar 24;11(2):e00028-20. doi: 10.1128/mBio.00028-20 (PMC7157512; doi:10.1128/mBio.00028-20)
Supplement: FIG S5 [file mBio.00028-20-sf005.docx]

**Suppl. Figure 5. Cross-neutralization PRNAs of VSV-ANDV escape mutants.** Clonal populations of each escape mutant virus in the presence of 128 IC_50_ of mAb were isolated via plaque picking of infected Vero cells. A stock of three clones of each virus was then grown, and PRNAs (and sequencing) were performed on these clones. Each graph **A**-**M** represents two experiments conducted on a single clone of the indicated escape mutant virus, with two technical replicates. All trend lines are logarithmic regressions except where such regressions were not converged; in these cases, connecting lines were used instead. Due to time and material constraints, PRNAs were not repeated with lower concentrations as in Figure 3. The negative control antibody in each case was consistent across the entire escape virus, but may vary between graphs: Each experiment used either KL-2G12 or KL-HA1-1D7.
